# Is passive leg raising clinically useful in predicting intradialytic hypotension?

**DOI:** 10.1186/s40635-024-00683-y

**Published:** 2024-11-19

**Authors:** Martin Ruste, Jean-Luc Fellahi, Matthias Jacquet-Lagrèze

**Affiliations:** 1 Service d’anesthésie-Réanimation, Hôpital Louis Pradel, Hospices Civils de Lyon; 59, Boulevard Pinel, 69677 Bron Cedex, France; 2 Faculté de Médecine Lyon Est, Université Claude Bernard Lyon 1, 8, Avenue Rockefeller, 69373 Lyon Cedex 08, France; 3 Laboratoire CarMeN, Inserm UMR 1060, Université Claude Bernard Lyon 1, Lyon, France

**Keywords:** Intradialytic hypotension, Net ultrafiltration, Passive leg raising, Diagnostic study

## Correspondence

We read with great interest the recent research article by da Hora Passos et al. [1], aiming at predicting intradialytic hypotension during intermittent haemodialysis with net ultrafiltration in critically ill patients. The authors are to be commended for exploring a cornerstone in the management of patients with acute kidney injury and fluid overload, where strong evidence-based strategies are lacking. However, we would like to discuss the method used here to assess the “clinical utility” of passive leg raising test (PLR) and dynamic arterial elastance “as predictors” of intradialytic hypotension.

In part, the authors conducted the study as the development of a clinical prediction model using logistic regression to identify variables associated with intradialytic hypotension. First, the number of events-per-variable in the model and the method used for the selection of variables could be criticised [2], although often observed in the scientific medical literature. However, the aim of the study was not to provide a multiparametric clinical prediction model, but to specifically analyse the prediction performance of both PLR and dynamic arterial elastance. As the latter is not significantly associated with intradialytic hypotension in the multivariate analysis, the authors focused on PLR. In this context, PLR could be considered as a diagnostic test to predict an outcome (the intradialytic hypotension). If a positive PLR before haemodialysis with net ultrafiltration would accurately predict the occurrence of intradialytic hypotension, the attending physician could adjust haemodialysis settings, patient monitoring, and vasoactive drugs to prevent haemodynamic intolerance of intermittent haemodialysis with net ultrafiltration. Such a methodology falls within the STARD 2015 guidelines for reporting accuracy studies [3]. Following this statement, authors are encouraged to report the discrimination performance of PLR (the index test) to predict intradialytic hypotension (target condition) using the area under the receiver operating curve, and to assess the best threshold of the variation in cardiac index/stroke volume during PLR with its sensitivity and specificity. Such an approach could confirm (or not) the relevance of PLR to be used as a guiding parameter to promote haemodynamic tolerance of haemodialysis in a population with lower net filtration rate than previously described [4]. A complete checklist with the whole items of the STARD statement could be used during the submission process for methodological guidance.

In our opinion, this would be a better way to assess the clinical utility of PLR in predicting intradialytic hypotension, rather than providing a survival univariate analysis that might suggest that fluid responsiveness prior to initiation of intermittent haemodialysis might per se be associated with mortality in critically ill patients.

## Data Availability

Not applicable.
